# Progressive Traffic-Oriented Resource Management for Reducing Network Congestion in Edge Computing

**DOI:** 10.3390/e23050532

**Published:** 2021-04-26

**Authors:** Won-Suk Kim

**Affiliations:** Department of Multimedia Engineering, Andong National University, Andong 36729, Korea; wonsukkim@anu.ac.kr; Tel.: +82-54-820-7968

**Keywords:** edge computing, fog computing, Internet of Things, cloud computing, network management, network architecture, software defined networking, heuristic algorithm

## Abstract

Edge computing can deliver network services with low latency and real-time processing by providing cloud services at the network edge. Edge computing has a number of advantages such as low latency, locality, and network traffic distribution, but the associated resource management has become a significant challenge because of its inherent hierarchical, distributed, and heterogeneous nature. Various cloud-based network services such as crowd sensing, hierarchical deep learning systems, and cloud gaming each have their own traffic patterns and computing requirements. To provide a satisfactory user experience for these services, resource management that comprehensively considers service diversity, client usage patterns, and network performance indicators is required. In this study, an algorithm that simultaneously considers computing resources and network traffic load when deploying servers that provide edge services is proposed. The proposed algorithm generates candidate deployments based on factors that affect traffic load, such as the number of servers, server location, and client mapping according to service characteristics and usage. A final deployment plan is then established using a partial vector bin packing scheme that considers both the generated traffic and computing resources in the network. The proposed algorithm is evaluated using several simulations that consider actual network service and device characteristics.

## 1. Introduction

Edge computing is a paradigm proposed to address various issues encountered when network services operate based on cloud computing [1,2]. In this concept, some of the resources such as computing, storage, and network that were previously provided in cloud data centers are provided at the edge of the network close to the users or things that use them. In other words, part of the role of the data center is to move some geographically dispersed physical equipment (including network equipment) such as switches, Wi-Fi access points, and telecommunication base stations close to users [3,4]. Therefore, if cloud computing is supplemented with edge computing, it is possible to retain all the advantages of a cloud system while reducing network latency, supporting real-time services, and providing services that reflect the geographical characteristics.

The main advantages of edge computing are low latency, location awareness, mobility support, and device heterogeneity [5]. Autonomous driving is a good example of services based on edge computing. This service can make good use of the location awareness feature of edge computing, which can provide traffic or area-related information collected locally to the autonomous driving system and notify it of road events such as construction or accidents [6]. Information can also be delivered to the driver or occupant using augmented reality on a head-up display on the windshield of the vehicle. In this scenario, gaze tracking of the driver or passenger, video processing, and object detection may be processed using the computing resources of adjacent edge devices [7,8]. This is possible because the edge computing model has significantly lower latency than the cloud model. Big data processing such as smart city disaster monitoring systems are another good example. The recognition of specific disaster patterns can be performed over the long term through data collection from geographically dispersed sensors in cloud data centers. In contrast, functions that require a rapid response, such as actuator operation according to pattern matching, can be configured to be performed quickly in the edge device [9,10].

Because edge computing is increasingly recognized as a next-generation model and questions about its feasibility can now be resolved, certain services are being refocused. Cloud games, which are in the introduction stage, are being reexamined because of the spread of infectious diseases and hardware limitations of game consoles. To overcome the limitations of the client hardware, a cloud game collects user inputs from the client, processes the collected information, and renders the graphics accordingly in the cloud, and then streams only the rendered frames to the client. In the cloud computing model, cloud games were unfortunately difficult to realize because of the round-trip time, but in the edge computing environment, the problem of latency is solved, making it a potentially feasible service [11].

### 1.1. Motivations

Edge computing has many advantages, but it is still applied only in limited scenarios because of the many constraints on its feasibility. Currently, telecommunication systems support edge computing in the form of multi-access edge computing within the core network. To bring edge computing to general-purpose Internet infrastructure such as local area networks (LANs), there are various issues that must be addressed.

First, edge computing processes a variety of services in edge devices close to users, and these devices have less powerful computing resources than the hardware of a cloud system. It is difficult to process multiple services in one node, and therefore, it is essential to consider geographically distributed computing. In addition, the environment in which edge computing operates, such as a LAN, is not exclusively dedicated to this model. In contrast, edge computing must coexist with other Internet service traffic. That is, server instances of services using edge computing need to be deployed according to the computing resources of the edge device and the state of the network.

Second, it is necessary to consider different resource requirements depending on the service. For example, a monitoring service that collects data from sensors requires relatively few computing resources. In contrast, services that perform video processing or algorithms with high complexity use a large amount of CPU and memory resources because they have a relatively large amount of computation. Cache and database servers require fewer CPU and memory resources but have relatively high storage requirements because they need to store a lot of data. When server instances of these services are deployed, if many servers that use a lot of one specific resource are deployed on one edge device, the other resources of the device may remain unused and hence wasted. In addition, a quick response to new resource factors that could occur in future is required.

Third, services based on cloud games, cloud virtual reality, and augmented reality, which are next-generation content platforms, may have a different level of traffic from previous ones [12,13,14]. This means that edge computing should be able to respond fairly sensitively to network traffic. A service that utilizes edge computing can place its own server instance in the local network, which means that traffic usage in the local network will change significantly depending on the location of the server deployed on the edge device. Reducing the traffic load in the network to prevent congestion will also improve the low-latency features of edge computing.

Fourth, in the local network where cloud-based services exist, most of the traffic exists between the gateway and specific nodes. However, in a network in which edge computing is applied, most of the traffic will occur between the nodes to which clients are connected and the node where the edge server of their services is located. In addition, to reduce the traffic between the server and the client, there may be more than one server instance for a particular service if the implementation allows it.

### 1.2. Contributions

In this study, a server instance placement algorithm is proposed that reflects the above considerations. It also takes into account the various computing resource requirements of services and resource capacity of edge devices while maintaining a low level of congestion in the network. The proposed algorithm operates by configuring node candidates in which a server for each service can be placed, and then selecting the candidate that optimizes computing resource use and network traffic load. Because the proposed algorithm simultaneously considers computing resources and network traffic, it facilitates the management of network performance indicators in edge computing.

In detail, this technique has the following contributions:

- The proposed scheme supports a service that uses two or more servers to cope with edge devices with relatively few computing resources.
- Because a vector bin packing (VBP) scheme is used, it is possible to increase the efficiency of computing resource use in the network even when different services request various types of resources.
- When clients use multiple services at the same time, it is possible to provide a quick service response time and reduce the traffic load by deploying the server as close as possible to the client while considering computing resources.
- For network congestion control, the proposed method includes a specific method for determining the priority for each candidate by estimating the number of servers and the traffic generated according to the client connections allocated for individual services. This method then selects the most appropriate candidate in the final plan.

## 2. Related Work

With the spread of Internet of Things (IoT) services, the concept of cloud computing has naturally expanded. In recent years, the concept of edge computing was established, and research related to edge computing has since been actively conducted. The existence of several similar technologies, such as fog computing and cloudlets, means that there is not yet a standardized or unified form of operation in this field. Paradoxically, many topics in this field are still key research subjects as a result. When the concept was first presented, studies were mainly conducted on usability and application services. Subsequently, research on computing and network resource management or architecture is now actively progressing.

Hu et al. [15] implemented a facial recognition system based on the fog computing environment. In this system, after analyzing the image transmitted from the fog node adjacent to the user, the extracted face identifier is transmitted to the cloud. By taking computational work from the cloud and distributing it to fog nodes, the overall processing efficiency was improved, and network transmission was reduced. Xu et al. [16] proposed an automatic fog server deployment framework that analyzes the packets for service requests and retrieves and installs the necessary applications from the repository. These studies proposed fog device platforms that support various services, but the platforms deploy a fog server only for a single user connected to the fog device and do not consider computing resources.

Bellavista et al. [17] proposed a method to build real fog middleware using the message queuing telemetry transport (MQTT) protocol in a fog computing environment. The middleware facilitates service interoperability and portability by configuring the service in the form of a Docker container. In addition, it enables the execution of multiple containers on a resource-constrained node such as the Raspberry Pi and has excellent scalability. Mahmud et al. [18] proposed a latency-aware application module management policy aiming at quality of service (QoS), application program optimization, and resource optimization based on the deadline. A method of optimizing the number of resources without violating the QoS of the application was also introduced. In this study, it was mentioned that if a server instance on a specific fog node is relocated to another fog node, the corresponding node can be turned off. However, in general, a fog server operates on a network device, so it is not necessary to turn off the device even if there is no server in operation. In addition, if the server is moved without considering traffic, the possibility of network congestion due to a service that generates a lot of traffic such as cloud games is very high.

In the field of edge computing, studies on service implementation based on the edge computing concept, studies on edge nodes (i.e., physical equipment including network nodes), and migration studies on the application of edge computing have been conducted. In addition to these topics, research on how to deploy which server to which edge node for which purpose is also essential from the network perspective.

The deployment of virtual machines through VBP algorithms in a cloud data center has been actively investigated. For instance, Shi et al. [19] proposed a method of placing virtual machines in a physical machine using a VBP algorithm in a data center. A technique was proposed to reduce the power consumption of the data center by reducing the number of physical machines on which virtual machines are placed through packing. In addition, various packing algorithms that can be used for virtual machine deployment were compared. The findings of [19] cannot be directly applied to edge computing because the study did not consider various factors that should be considered in edge computing. Moreover, an edge network topology was not considered, which limits the study’s relevance to edge computing. An edge node is characterized by fewer computing resources than equipment in a data center, and recent network services utilize not only CPU and memory resources, but also storage, GPU, and other resources, so it is essential to consider the various types of computing resources required.

Song et al. [20] studied a technique for deploying virtual machines in a data center by expanding the bin packing problem in multiple dimensions. An algorithm for bin packing items with variable sizes was defined, expanded to three dimensions, and compared with other algorithms. However, there is a clear difference between handling virtual machines within the data center and managing server instances at the edge of the network in terms of the environment and the purpose of the algorithm.

In research on data centers, the focus is on how few physical machines can be used to provide the same service based on the required resource information of the virtual machines. This is because a physical machine that is not in operation can reduce power consumption by entering sleep mode, and this simplifies operations. However, in an edge computing environment, minimizing the number of nodes operating at the network edge becomes a rather trivial problem. The focus of edge computing research is to minimize the response time to clients, sensors, data sources, and the entities from adjacent networks. In other words, handling various factors in the network that affect the key performance indicator of network latency is more important than how few physical machines are used. These factors include not only computing resources, but also network congestion control and even the traditional hop count indicators of distance to the requester.

Wang et al. [21] and Li et al. [22] studied an optimization problem for deploying edge servers in a mobile edge computing environment. Reference [21] has formulated a multi-objective constraint optimization problem for determining the edge server location to balance the workload between edge servers and minimize edge server access latency. Reference [22] focused on minimizing the total energy consumption of edge servers while keeping access latency at a reasonable level. In both studies, the optimal solution to the problem was derived through a mixed integer programming. These studies have suggested an approach to minimizing access latency or workloads when the workload offloading relationship between base stations and edge servers in a mobile network environment constitutes a many-to-one relationship. However, these studies are vulnerable to network service diversity, and have limitations in that the traffic flows related to the edge server are simplified into a many-to-one relationship between base stations and server.

Mukherjee et al. [23,24] studied a fog computing instance in terms of end-user latency. For a specific task of the user with a specified acceptable latency, if it seems that the latency cannot be satisfied in the local system by lack of computing resources, the task is offloaded to the primary fog node in the primary coverage. By this offloading, the transmission latency slightly increases, but the total latency is reduced by decreasing the computing latency by utilizing relatively sufficient available computing resources. If it is determined that the acceptable latency of the task cannot be satisfied with the computing resources of the primary fog node, a part of the task is offloaded to the cloud server or the secondary fog node while additionally sacrificing the transmission latency. Through this approach, the acceptable latency experienced by the users is guaranteed as much as possible. These studies have contributed to a more specific approach to computing resources such as CPU cycles. However, the proposed offloading strategy has a limitation in that it focuses only on the total latency of individual users.

In our previous study [25], an algorithm for deploying servers in an edge computing environment was presented. The servers were first deployed using the hop count between the client and the server instance as the main metric, and then the VBP algorithm was used heuristically to relocate a server with low priority to another node. Conceptually, reference [25] presented an algorithm that combines computing resources and network metrics, but there are several limitations that prevent practical application.

First, the network traffic load was excluded in the service flow concept presented in the previous study. The service flow refers to a set of flows formed between several clients using a specific service and the edge server in the network that provides the service. Only computing resources were considered in the usage amount for specific services, and network metrics such as traffic load were not used. Determining network resources using only the hop count means that network congestion is likely because bottlenecks can occur on certain paths. Second, the division of clients in the service flow is ambiguous. The algorithm is limited because it assumed that all clients using a specific service have the same usage type. It moreover assumed that one client uses only one service. Even for clients using the same service, there are obvious differences in usage patterns. Third, it assumed that a service has only one server in the network. In an actual service, its server can be composed of multiple mirror servers, cache servers, and hierarchical servers [26].

In this study, a server deployment and management scheme that is closer to practical application is proposed by additionally considering differing client usage patterns and the network traffic load. Furthermore, an algorithm for determining the optimal server operation type from the viewpoint of network congestion control is proposed.

## 3. Main Algorithms

**Figure 1:**
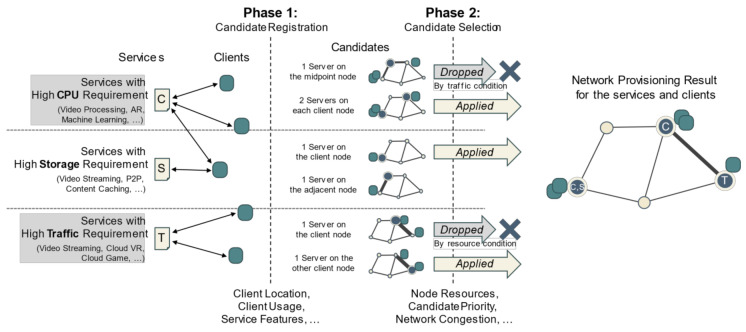
Proposed candidate-based configuration algorithm for a network in which edge computing is used. Phase 1: candidates are created and registered based on the features of each service as well as the location and usage of clients. Phase 2: candidates are selected for services considering node resources and traffic load.

The primary purpose of the proposed algorithm is to simultaneously achieve the efficient use of computing resources and minimization of network traffic. Further, it presents a method for simultaneously considering two different performance indicators with different characteristics. Our algorithm, as shown in Figure 1, consists of two steps. The first step determines candidate nodes for server deployment for each service according to the expected traffic. The next step selects the most appropriate candidates to optimize the computing resource use and network congestion. In actual application, the balance between resource efficiency and network congestion can be easily adjusted when configuring a server according to the state of the network.

### 3.1. Basic Components

The proposed edge network configuration algorithm is based on an edge computing environment operating in a local network of a certain size. This subsection describes the basic characteristics of and terms that refer to network components for the proposed algorithm.

First, the service collectively refers to the Internet services, applications, and IoT services that operate using the cloud and edge computing environments. The communication of a service can include intermittent synchronization, real-time synchronization, file transfer, REST application programming interface, or authentication communication, but in the proposed algorithm, we consider only traditional server-client model communication. This does not include peer-to-peer file transfer but does include cloud-based applications such as cloud games.

In the edge server (or server) of the service, various tasks can be performed according to the type of service. A task requires specific computing resources such as CPUs, memory, storage, and GPUs depending on the operation. For example, remote video processing services have a high CPU load, database services have high memory load, and cloud storage services have high storage load. In addition, the traffic load generated for each client is different depending on the operation type. A crowd sensing service has low or moderate traffic per unit time, but a cloud game or cloud virtual reality service can have very high traffic. Each service has various computing resource loads and traffic load according to the client usage. The client usage (or usage) is a concept that can be expressed in various forms, for example, time used per day or traffic generated per unit time.

The edge node (or node) refers to a device in which the server of the service actually operates. The edge node can be any computing device located on the edge network. This can be a network device equipped with computing functions such as a Wi-Fi access point, hub, switch, IoT gateway, roadside unit, or mobile base station. Alternatively, it can be a computing device directly connected to a router such as a cache server or general-purpose physical machine. From a broad perspective, it may even include user handheld devices such as smartphones or even sensor nodes. In this study, to ensure the description of the algorithm is brief, the range of edge nodes is limited to network devices or computing devices directly connected to the network device. It can be seen that such an assumption does not deviate significantly from the actual application, as in the case of multi-access edge computing.

Each service deploys its own server as a container within the edge node. This is made possible through network technologies such as software-defined networking and container technologies such as Docker [27,28,29,30]. In this study, it is assumed that each node has its own computing resource capacity, and the server is deployed such that the available resources of the edge node are not exceeded.

For the service to operate the server on the edge network, several things must be considered. It should be determined how many servers should be operated to process client requests, the relationship between the servers (if more than one is operated), and what happens to base operations other than the core operation (e.g., database operations). In this study, the above factors are abstracted using the number of servers and client usage. It is assumed that the service already knows the CPU, memory, storage, and traffic loads for the usage of each client.

Operations such as database, firewall, and system call operations that are required to operate the server are called based operations. A resource required by the base operation is called the base resource. This resource is required to run the server, so its resource load does not depend on client usage. The base resource also includes resources such as the minimum CPU and memory resources allocated to the server container. This is one of the factors to consider when running servers that take on the same role in multiple nodes.

In this study, when more than one server is used, it is assumed that they operate only in the form of clones. Synchronization for information exchange may also be required in this form (although it is not for active collaboration). Traffic for this purpose is called synchronization traffic. The total computing resource load and network traffic load of the service are determined by the total usage of the clients using the service. In addition, there is a synchronization traffic load among servers and base resources that are not dependent on the usage.

### 3.2. Phase 1: Candidate Registration by Network Traffic

To support edge computing, an administrator of an edge network should place edge servers on the appropriate edge nodes and associate them with specific clients. It is assumed that the administrator knows the available resources of all edge nodes within his or her management range and can secure the service usage for each client.

There are two main factors to consider when placing servers on the nodes: computing resources and networking resources. The computing resources are independent for each node, but the networking resources are interrelated from a network-wide perspective. In addition, computing resources are more often constrained than networking resources. Therefore, it is not appropriate to consider network resources as a same level with computing resources in a multi-dimensional VBP algorithm that allocates as many items as possible to a specific bin without overlapping. To consider network resources, preprocessing must be performed before VBP. This preprocessing is the process of registering candidates for server deployment per service.

A candidate for server deployment is expressed as a combination of clients using a specific service and servers that can be deployed on the network. In more detail, it is a combination of cases in which the servers are deployed to specific nodes without overlapping to provide services to the clients. The signature of a candidate is composed of the number of servers, nodes on which each server is assumed to be deployed, and mapping information for each server and client. For a specific service, the following examples can be candidates: one server is deployed on node A to provide service to clients X and Y, one server is deployed on node B to provide service to clients X and Y, two servers are deployed on nodes A and C so that the first server provides service to client X and the second server to client Y, and in the same situation, the first server provides service to client Y and the second server provides service to client X.

Computing resources are not considered in the candidate registration process: only the traffic load is considered. Each candidate includes the total generated traffic load when the candidate is actually applied, and registered candidates are sorted in ascending order based on the expected total traffic. That is, given the service characteristics and the usage of the clients, the candidate generating the least traffic according to the number and location of servers is the prime candidate for the service. This server deployment candidate registration process is detailed in Algorithm 1.

The meanings of symbols used in the algorithms are shown in Table 1.

Algorithm 1 is the process of registering server deployment candidates for a specific service v. The input of the algorithm consists of a network service, a set of nodes including topology information, and the set of clients using the service. The output is a list of server deployment candidates for the input service, and this list is sorted in ascending order according to the amount of generated traffic.

In line 6, an empty candidate list C is first created. In line 7, candidates are generated by sequentially increasing the number of expected servers of the service by one. In other words, candidates are registered while increasing the number of servers to be considered; for example, candidates composed of one server are created and registered, and then candidates composed of two servers are registered. This process is repeated until the number of servers to be considered reaches the maximum number of clients or the number of nodes (whichever is smaller). Here, $\left|C\right|$ is the number of clients using service v. As described later, it is rare that a large number of servers are configured when creating a candidate, so only the case consisting of one to four servers on average is considered. In line 8, the expected server set $S_{\lambda }$ when there are λ servers for service v is generated. At least one client is connected to each server in this set. This set is passed as a parameter to the *GetServerAssignment* submodule along with the node information in line 9.

*GetServerAssignment* computes and returns information about the server assignment. The term server assignment refers to each case in which each server of the same service can be deployed without overlapping nodes in the network. The servers are not distinguished at this stage. This assignment can be represented as a set of key-value pairs, where the key is the server, and the value is the node. For example, if two servers $s_{1}$ and $s_{2}$ are deployed without overlapping on nodes $n_{1}$, $n_{2}$, and $n_{3}$, the server assignment will be ($s_{1}:n_{1}$, $s_{2}:n_{2}$), ($s_{1}:n_{1}$, $s_{2}:n_{3}$), ($s_{1}:n_{2}$, $s_{2}:n_{3}$). The elements enclosed in parentheses are the information for an individual assignment, and in the example above, three assignment cases are created. The number of generated assignments is $\left(\begin{array}{c} \left|N\right|\\ \lambda \end{array}\right)$.
**Algorithm 1.** Registration of Candidate for Server Placement of a Certain Service1Input:2v← an input service3N← the set of edge nodes4C← the set of clients using the service v5
6C← set as empty list for the server placement candidates for service v7**for**$\lambda \leftarrow \left[1,\min\left(\left|N\right|,\left|C\right|\right)\right]$**do**8         $S_{\lambda }\leftarrow$ the set of servers when service v has λ of servers9         $A_{\lambda }\leftarrow GetServerAssignment\left(N,S_{\lambda }\right)$
10        // The set of assignments for λ of servers $A_{\lambda }$ consists of sets of key-value pairs11        // key: a server, value: a node running the server12        // i.e., $A_{\lambda }=\left\{\left\{s_{1}:n_{5},s_{2}:n_{7},s_{3}:n_{2},\ldots \right\},\;\left\{\ldots \right\},\;\ldots \right\},\;\forall s_{i}\in S_{\lambda },\forall n_{j}\in N$
13        **for each**
$a\in A_{\lambda }$
**do**14                $\tau_{a}^{sync}\leftarrow GetSyncTraffic\left(v,\;a\right)$
15                $M_{a}\leftarrow GetMappingInfo\left(C,\;a\right)$
16                // The set of mapping information for assignment a, $M_{a}$ consists of sets of key-value pairs17                // key: a client, value: a server mapped with the client18                // i.e., $M_{a}=\left\{\left\{c_{1}:s_{2},c_{2}:s_{2},c_{3}:s_{1},\ldots \right\},\;\left\{\ldots \right\},\;\ldots \right\},\forall c_{i}\in C,\;\forall s_{j}\in S_{\lambda }$
19                **for each**
$m\in M_{a}$
**do**20                        $r_{m}^{base}\leftarrow GetBaseResource\left(v\right)$
21                        $\tau_{m}\leftarrow \tau_{a}^{sync}$
22                        **for each**
c∈m.Keys
**do**23                                $s_{c}\leftarrow$ the server mapped with client c24                                $\tau_{m}\leftarrow \tau_{m}+u_{c}\times f_{t}^{v}\times dist\left(s_{c},\;c\right)$25                                $r_{m}^{s_{c}}\leftarrow r_{m}^{s_{c}}+u_{c}\times \left[f_{cpu}^{v},\;f_{mem}^{v},\;f_{sto}^{v}\right]$
26                        **end for**27                        **for each**
$s\in S_{\lambda }$
**do**28                                $r_{m}^{s}\leftarrow r_{m}^{base}+r_{m}^{s}$
29                                $R_{m}.Add\left(r_{m}^{s}\right)$
30                        **end for**31                        ${𝕔}_{\lambda }^{m}\leftarrow CreateCandidate\left(a,m,\;\tau_{m},R_{m}\right)$
32                        $C.Register\left({𝕔}_{\lambda }^{m}\right)$
33                **end for**34        **end for**35        $C.Sort\left(\tau ,ascending\right)$
36        **if**
$\lambda >1\;\wedge \;∄{𝕔}_{\lambda }^{m}\in C,\;\;{𝕔}_{\lambda }^{m}.\tau <{𝕔}_{\lambda -1}^{m}.\tau$
**then break**37**end for**38
39Output:40**return**C

Beginning on line 13, the operation of mapping client information to each assignment in the assignment set is performed. The *GetSyncTraffic* submodule on line 14 receives service characteristics and assignment information and calculates the traffic required for synchronization among all the servers in the assignment. The synchronization traffic is determined by the service characteristics and the location of the servers. For example, content streaming services will have low levels of synchronization traffic, and services that require real-time collaboration between servers such as cloud gaming will have very high levels. It is assumed that this synchronization traffic factor is already included in the service-specific characteristics. To reduce complexity, it is further assumed that the synchronization traffic load does not depend on the client usage. To calculate the synchronization traffic, a graph is created. The gateway node and each node on which the server is deployed form the vertices and the number of hops between servers is the weight of each edge. Next, the minimum spanning tree of the graph is calculated, and the value obtained by multiplying the weight of each edge in the tree by the synchronization traffic factor of the service characteristics becomes the synchronization traffic load for that assignment.

In line 15, client mapping information is calculated for each assignment. *GetMappingInfo* uses the assignment and client information to obtain the connection between the server and client. The mapping is a set of key-value pairs indicating which client is connected to which server for a specific assignment. There can be multiple mapping results in a single assignment. The server at this stage is the server in the assignment, and each server is uniquely treated because it is deployed on a specific node. Because a specific client must be connected to one server, the mapping operation becomes a problem of permutations with repetition (or n-tuples) that selects as many servers as the number of clients in order while allowing redundancy. The number of permutations is $\lambda^{\left|C\right|}$. For example, assume that the assignment a consists of two servers $s_{1}$ and $s_{2}$ located on different nodes, and there are three clients $c_{1}$, $c_{2}$, and $c_{3}$. Since the number of servers λ is 2 and $\left|C\right|$ is 3, the total number of mappings for the assignment a is 8. The mapping set $M_{a}$ results in $\left\{\left\{c_{1}:s_{1},\;c_{2}:s_{1},c_{3}:s_{1}\right\}\right.$, $\left\{c_{1}:s_{1},\;c_{2}:s_{1},c_{3}:s_{2}\right\}$, $\left\{c_{1}:s_{1},\;c_{2}:s_{2},c_{3}:s_{1}\right\}$, $\left\{c_{1}:s_{1},\;c_{2}:s_{2},c_{3}:s_{2}\right\}$, $\left\{c_{1}:s_{2},\;c_{2}:s_{1},c_{3}:s_{1}\right\}$, $\left\{c_{1}:s_{2},\;c_{2}:s_{1},c_{3}:s_{2}\right\}$, $\left\{c_{1}:s_{2},\;c_{2}:s_{2},c_{3}:s_{1}\right\}$, $\left.\left\{c_{1}:s_{2},\;c_{2}:s_{2},c_{3}:s_{2}\right\}\right\}$.

It is necessary to address the total number of mappings of the service. Unfortunately, if there are no restrictions, the total number of mappings becomes $\overset{\min\left(\left|C\right|,\left|N\right|\right)}{\underset{\lambda =1}{\sum }}\left(\begin{array}{c} \left|N\right|\\ \lambda \end{array}\right)\cdot \lambda^{\left|C\right|}$, which is obviously a very large number. To address this, it is necessary to put some restrictions on the connections between the server and the client. One restriction is that there is no reason for a server with no connected clients to exist because the algorithm searches for candidates while increasing the number of servers. Because at least one client must be connected to a server, the number of mappings is reduced considerably to $\overset{\lambda }{\underset{i=1}{\sum }}\left(\begin{array}{c} \lambda \\ i \end{array}\right)\cdot i^{\left|C\right|}\cdot {\left(-1\right)}^{\lambda -i}$. Considering the above example again, the mapping set $M_{a}$ becomes $\left\{\left\{c_{1}:s_{1},\;c_{2}:s_{1},c_{3}:s_{2}\right\}\right.$, $\left\{c_{1}:s_{1},\;c_{2}:s_{2},c_{3}:s_{1}\right\}$, $\left\{c_{1}:s_{1},\;c_{2}:s_{2},c_{3}:s_{2}\right\}$, $\left\{c_{1}:s_{2},\;c_{2}:s_{1},c_{3}:s_{1}\right\}$, $\left\{c_{1}:s_{2},\;c_{2}:s_{1},c_{3}:s_{2}\right\}$, $\left.\left\{c_{1}:s_{2},\;c_{2}:s_{2},c_{3}:s_{1}\right\}\right\}$. The number of mappings is $2\cdot 1\cdot \left(-1\right)+1\cdot 8\cdot 1=6$.

Unfortunately, the result of the above operation can also be very large in some situations. Additional constraints are required to lower the total number of mappings to a more reasonable number. For example, suppose there are nodes $n_{1}$ and $n_{2}$ that are physically distant in the network. Furthermore, suppose that servers $s_{1}$ and $s_{2}$ of a specific service are deployed on nodes $n_{1}$ and $n_{2}$, respectively, and clients $c_{1}$ and $c_{2}$ are also connected to nodes $n_{1}$ and $n_{2}$, respectively. In this case, it makes sense for client $c_{1}$ to connect with server $s_{1}$ and client $c_{2}$ to connect with server $s_{2}$. The reverse connection for any reason, including constraints from computing resources, has irrational consequences. To resolve the resource constraints, it makes more sense to adjust the server location rather than make a reverse mapping. Therefore, a client is mapped so that it is connected with the closest server in the assignment. As a result, there is only one mapping in the assignment if all clients have a unique closest server.

To secure some diversity in the candidates, when there are two or more servers that are same distance from a specific client, each server selection process for one client is treated as a different mapping. For each client, servers at the same distance are configured as a set, and all the Cartesian product results of the set of each client are used as mappings. For example, when four clients have one, two, one, and three servers at the same distance, respectively, the maximum number of mappings generated for the assignment is six. If there is a server that does not have any clients connected in the mappings created by the above process, this server is removed because of the previous constraints. Consider the example above again. Suppose that the distance between $c_{1}$ and $s_{1}$ is 3, $dist\left(c_{1},s_{2}\right)=2$, $dist\left(c_{2},s_{1}\right)=1$, $dist\left(c_{2},s_{2}\right)=1$, $dist\left(c_{3},s_{1}\right)=2$, and $dist\left(c_{3},s_{2}\right)=2$. The final mapping set is $\left\{\left\{c_{1}:s_{2},\;c_{2}:s_{1},c_{3}:s_{1}\right\}\right.$, $\left\{c_{1}:s_{2},\;c_{2}:s_{1},c_{3}:s_{2}\right\}$, $\left\{c_{1}:s_{2},\;c_{2}:s_{2},c_{3}:s_{1}\right\}$, $\left.\left\{c_{1}:s_{2},\;c_{2}:s_{2},c_{3}:s_{2}\right\}\right\}$. The number of mappings is 1·2·2−1=3. The last element is removed due to the constraint that the server should be connected with at least one client.

The total number of mappings for a specific assignment can be greatly reduced by the following constraints: (1) All clients select the nearest server among several servers in the assignment. (2) If a specific client has two or more nearest servers, a server set for that client is composed of those servers. Then, each element of the cartesian product for that server set of all clients becomes each mapping. (3) The mapping where the server to which the client is not connected exists is removed.

In lines 19 to 33, candidates are formed by adding metadata such as total traffic load and computing resources to each mapping. In line 20, the base resource for service v is obtained from the *GetBaseResource* module. This is later added to the required computing resources for each server. In line 21, the expected total traffic load for the mapping is initialized as synchronization traffic. In lines 22 to 26, the computing resource requirements of each server and the traffic load generated from the mapping are calculated for each client. The generated traffic can be obtained by multiplying the usage of each client by the traffic factor of the service and the distance to the server connected to the client. In this way, the expected traffic load for each client is calculated and added to the total expected traffic load for this mapping. In addition, the resource requirements for the server are calculated by multiplying the CPU, memory, and storage factors of the service for the usage of each client. From lines 27 to 30, the total resource requirements for each server are calculated by adding the base resource to the resource requirements for each server. In lines 31 and 32, the candidate is created and registered in the candidate list based on the assignment, mapping, expected total traffic, and required computing resources.

In line 35, after all candidates with λ servers are registered, the candidates are sorted in ascending order based on the expected traffic load. In line 36, it is determined whether additional candidates with an increased number of servers should be generated. When there are two or more servers, candidates with λ + 1 servers are not considered if none of the candidates with λ servers have a lower total traffic than any candidates generated with λ−1 servers.

This process completes the candidate list for service v. Each candidate contains information about a situation in which each client is connected to a nearby server when several servers are placed in the network without overlapping nodes. In the next stage, the final candidate is selected from among the registered candidates for each service.

### 3.3. Candidate Selection Using Partial VBP

**Figure 2:**
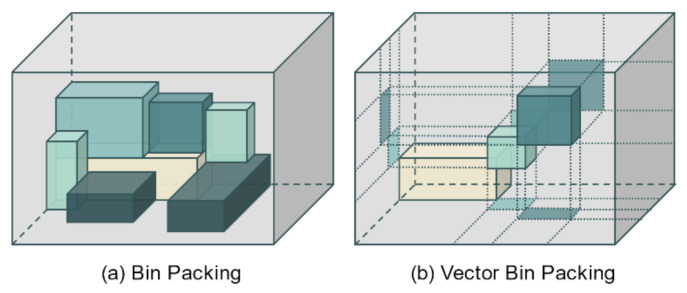
Differences between the common bin packing problem (**a**) and the VBP problem (**b**) used in this study.

After candidate registration for all services has been completed, for each service, it is next necessary to select the candidate that is actually deployed in the network. This may be abstracted as a problem of putting items of various shapes into as few bins as possible, that is, it can be seen as a part of the bin packing problem [31]. As shown in Figure 2a, only the size and shape of items and bins are considered in the general bin packing problem. However, because computing resources cannot be occupied by multiple processes at the same time, the VBP algorithm is used instead.

VBP is a widely used solution when deploying virtual machines in data centers [32,33]. As shown in Figure 2b, the aim of VBP is to deploy virtual machines so that the sum of the resource requirements of the virtual machines does not exceed the available resources of the physical machine. In particular, it is usually used to minimize the number of physical machines used. Compared with the normal bin packing in Figure 2a, it can be seen that when an item enters the bin, any axis occupied by a specific item is not occupied by overlapping with other items. Based on VBP, a virtual machine with a resource requirement that is most similar to the remaining resource form of the physical machine is placed on the physical machine, and in this way, a minimal number of physical machines can be operated without wasting the resources of the physical machine. Considering the available resources of the machine as an n-dimensional vector and the resource requirements of the service as another vector, the dot product of the available resource vector and the required resource vector is compared. The service with the smallest vector angle is determined to have the most appropriate resource requirements for operation on that machine.

VBP can adapt to an increase in the number of resource types by increasing the dimensions of the vector. In this study, a three-dimensional vector is used because three computing resources are considered: CPU, memory, and storage. The proposed algorithm also focuses on network traffic but does not do this by using a four-dimensional vector that includes traffic. This is because the computing resources affect only the node where the server is located. In contrast, the network traffic affects several network entities between the client and server. Therefore, in this study, network traffic is not considered as a dimension of the VBP, but as an independent metric instead.

Because the candidate list for each service is sorted in ascending order according to the expected traffic load, the first candidate of each list can be regarded as the prime candidate of the corresponding service from the perspective of network traffic. It would be the best case if the prime candidates for each service can be deployed in the network. However, when the prime candidates for each server are included in the deployment plan, the total resources required by the servers deployed in a specific node may be greater than its available resources. This node then becomes a node that needs server reconfiguration. To reconfigure the node, a partial VBP (PVBP) is used to select the server to be removed. Then, all servers in the candidate that includes that server are also removed from the plan. The next candidate for the service is then added to the plan, and validation is performed again. The PVBP uses VBP with a separate metric when selecting a server for replacement. This process is shown in Algorithm 2.

Algorithm 2 shows the server deployment method based on PVBP. The input of this algorithm is the weighted value of the traffic to be considered together with the VBP result when the server is reconfigured. The output of the algorithm is the plan, i.e., the set of final candidates selected for each service. While the algorithm is being executed, each candidate is added to or eliminated from the plan, and one candidate per service is included in the final plan. Servers included in each candidate in the final plan are then created and placed on the corresponding nodes.

In lines 5 to 8, all services first add their prime candidate to the plan. In line 7, the *Apply* module algorithmically places the candidate in the plan. Because the candidate has the assignment and mapping information, this operation virtually places the candidate in a specific node based on the traffic and resource requirement of the servers in that candidate.
**Algorithm 2.** Candidate Selection by Partial Vector Bin Packing1Input:2ω← the weight for traffic as metric34P← the set of candidates of the final plan5**for each**v∈V**do**6         $𝕔\leftarrow v.C\left[0\right]$
7         $P.Apply\left(𝕔\right)$
8**end for**910**while**true**then**11        $n_{r}\leftarrow null$ // the reconfigure node12        **for each**
n∈N
**do**13                $\left[r_{n}^{cpu.req},r_{n}^{mem.req},r_{n}^{sto.req}\right]\leftarrow GetRequiredResources\left(n,P\right)$
14                **if**
$r_{n}^{cpu}<r_{n}^{cpu.req}\;\vee \;r_{n}^{mem}<r_{n}^{mem.req}\;\vee r_{n}^{sto}<r_{n}^{sto.req}$
**then**15                        $n_{r}\leftarrow n$
16                        **Break**17                **end if**18        **end for**19        **if**
$n_{r}=null$
**then break**20        $metric_{max}\leftarrow -\infty$
21        $\left[\mu_{a},\;\sigma_{a}\right]\leftarrow GetAngleStats\left(P\right)$
22        $\left[\mu_{\tau_{d}},\;\sigma_{\tau_{d}}\right]\leftarrow GetTrafficDiffStats\left(P,V\right)$
23        $r_{n}\leftarrow \left[r_{n_{r}}^{cpu},r_{n_{r}}^{mem},r_{n_{r}}^{sto}\right]$
24        **for each**
$s\in n_{r}.S$
**do**25                $r_{s}\leftarrow \left[r_{s}^{cpu},r_{s}^{mem},r_{s}^{sto}\right]$
26                $\alpha \leftarrow \cos^{-1}\left((r_{n}\cdot r_{s})/\left(\left|r_{n}\right|\left|r_{s}\right|\right)\right)$
27                ${𝕔}_{next}\leftarrow GetNextCandidate\left(s\right)$
28                **if**
${𝕔}_{next}=null$
**then continue**29                $\tau_{d}\leftarrow GetTrafficDiff\left({𝕔}_{s},{𝕔}_{next}\right)$
30                $\alpha^{z}\leftarrow \left(\alpha -\mu_{\alpha }\right)/\sigma_{\alpha }$
31                $\tau_{d}^{z}\leftarrow \left(\tau_{d}-\mu_{\tau_{d}}\right)/\sigma_{\tau_{d}}$
32                $metric\leftarrow \alpha^{z}\times \left(1-\omega \right)-\tau_{d}^{z}\times \omega$
33                **if**
$metric_{max}<metric$
**then**34                        $metric_{max}\leftarrow metric$
35                        $s_{r}\leftarrow s$
36                **end if**37        **end for**38        $P.Remove\left({𝕔}_{s_{r}}\right)$
39        ${𝕔}_{next}\leftarrow GetNextCandidate\left(s_{r}\right)$
40        **if**
${𝕔}_{next}=null$
**then break**41        **else then**$P.Apply\left({𝕔}_{next}\right)$
42        **end if**43**end while**44
45Output:46**return**valid ?P:∅

Beginning at line 10, the candidates are replaced until the plan is valid. In lines 11 to 18, the plan is validated by searching for any nodes that need reconfiguration. *GetRequiredResources* in line 13 returns the sum of the resource requirements of all servers allocated to that node. The total resource requirements are compared with the available resources of the node by type, and if there is any resource type that cannot be accommodated, the node becomes a reconfiguration node. If no reconfiguration nodes are found during this process, all servers can be deployed normally, and the final plan is returned.

If a reconfiguration node has been selected, an operation that replaces one of its servers is performed. Note that not just the server to be replaced, but all servers in the candidate that contains that server are removed from the plan. In lines 21 to 22, statistical calculations on the angle and the traffic difference are performed for all servers and nodes, and the values are retained. These values are then used for standardization, as described later (lines 30 to 31).

The metric score, which is the value for selecting a server to be removed from the reconfiguration node, is defined as $metric\leftarrow \alpha^{z}\times \left(1-\omega \right)-\tau_{d}^{z}\times \omega$. That is, the standardized vector angle and the traffic difference from the next candidate are considered together according to the weighted value. This metric was defined by referring to the well-arranged for metric design guideline in the IETF document [34]. The metric reflects the characteristics of the LAN well and separates computing resources and traffic load that have orthogonality from each other. It can also be applied to large-scale LANs. Details of the metric will be described in the following algorithm description.

Starting at line 23, metrics are calculated for each server in the reconfiguration node, and the server with the highest metric score is replaced. The vector of the reconfiguration node is calculated in line 23 and the vector of each server is calculated in line 25. The vector angles are then obtained using the dot product. Note that the purpose of server deployment and replacement using VBP is to use the computing resources of the node most efficiently. In other words, the use of VBP implies that the server with resource requirements that differ most from the available resources of a reconfiguration node is eliminated. Because the proposed algorithm considers three types of resources, a three-dimensional vector is generated, and the angle is calculated. However, as mentioned above, the reason that VBP is partially utilized is because the network traffic cannot be included in the VBP calculation.

From lines 27 to 29, the traffic difference $\tau_{d}$ is calculated, where ${𝕔}_{s}$ is the candidate to which the server belongs, and ${𝕔}_{next}$ is the next candidate in the service. The traffic difference is the difference in expected traffic between the ${𝕔}_{s}$ and ${𝕔}_{next}$. The purpose of using the traffic difference as a metric is to minimize the increase in traffic when a specific candidate is removed, and the next candidate is newly added. Therefore, unlike the vector angle, a smaller traffic difference leads to a higher replacement priority. This is the reason why the traffic difference is subtracted when the metric is calculated. However, if there is no next candidate in the service to which the server belongs, the server cannot be replaced and is therefore ignored.

To reduce unnecessary repetition of the plan validation process and employ the traffic difference more accurately, several issues should be considered when selecting the next candidate.

First, the method of selecting the next candidate should be considered. Because the candidate list of each service is sorted based on traffic, there is the simple method of selecting the candidate immediately following the selected candidate. A candidate that does not require additional computing resources for the reconfiguration node could also be selected. The former approach is rational in terms of the network traffic, and the latter is rational in terms of computing. However, the candidate registration process considers several nodes at the same time, and traffic is strongly affected by server location, so there is some correlation between the order of candidates and assignments. That is, because candidates adjacent to each other in order have similar assignment information, if only the next candidate is selected, the possibility that the reconfiguration node will need to be reconfigured again is very high. That is, the former method may validate the plan more times than necessary, so the proposed algorithm selects the next candidate using the latter method.

Second, the method of selecting a target candidate for calculating the traffic difference should be considered. There are two ways to do this: one is to evaluate the difference between the prime candidate and next candidate, and the other is to evaluate the difference between the current candidate and next candidate. In the former case, a service with an overwhelmingly low traffic load of the prime candidate has a very low possibility of replacement compared to other services. Because the traffic load of the candidate currently being considered becomes meaningless, it is not suitable as the metric. In the latter case, when the number of candidates increases and the traffic difference with the immediately next candidate decreases, the possibility of misleading the metric increases. Fortunately, the next candidate is likely to be a candidate in a slightly distant order, not just in order as described above. Therefore, since the misleading of the metric naturally decreases, it is reasonable to consider the traffic difference with respect to the current candidate to reflect the traffic increase most appropriately.

In lines 30 to 31, standardization is performed. Standardization must be performed to compare two different types of data: the computing resource vector angle and the traffic difference between the current and next candidate. Through standardization, the amount of change in the vector angle can be directly compared with the amount of change in traffic difference. A population of data is needed to calculate the average and standard deviation for standardization, and the vector angle population is the angles between all currently planned servers and the nodes where the servers are to be deployed. The population for the traffic difference is the differences between the candidate to which all currently planned servers belong and its next candidate. These statistical calculations are performed initially when planning the prime candidate deployment of all services. Subsequently, additional calculations only need to be performed for the servers in candidates to be removed or added.

In line 32, the score of the final metric of each server is calculated, and the balance between the vector angle and the traffic difference is determined by the traffic weight ω. The server with the highest score is the target of replacement, and a high score means that the vector angle is large, and the traffic difference is small. A high vector angle means that there is a large difference in the resource ratios of the server and the node, and a low traffic difference means that the impact on the network if the server is replaced is low. That is, PVBP is used so that, from among the candidates for which the server has a resource configuration that is significantly different from the reconfiguration node, the candidate whose replacement least influences the network is replaced.

In line 38, the candidate selected for replacement is removed from the plan, and in line 39, the next candidate is applied to the plan. If there is no suitable candidate, all service candidates related to the node have been exhausted, and the algorithm is considered to have failed.

Because the candidate includes the assignment information of multiple servers, replacing the candidate using PVBP affects the resource status of other nodes as well as the reconfiguration node. Therefore, when the candidate is replaced, resource validity should be rechecked for all nodes. If all the required resources for all nodes are acceptable, the plan is applied to the network.

### 3.4. PVBP Algorithm Example

**Figure 3:**
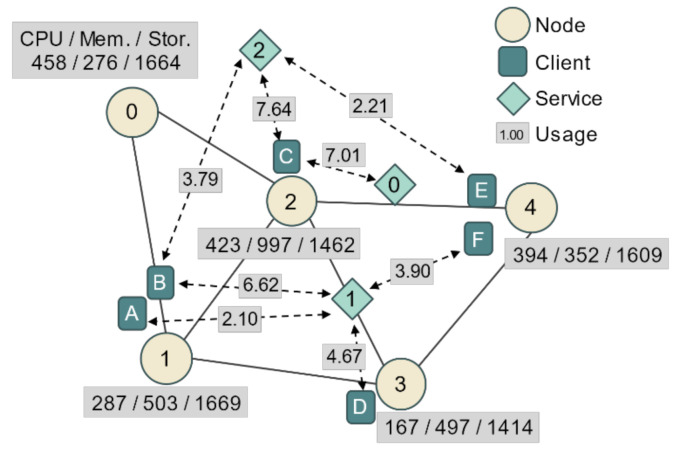
Example network topology with edge nodes, clients, and services.

In this subsection, a simple example is presented to demonstrate how the proposed algorithms work. Figure 3 shows the network topology used in the example. The connection information between nodes, available computing resources of the nodes, location of the clients, services used by the clients, and service usage of each client are shown. The clients are connected to the network through nodes, and the dotted arrows indicate the services used by the clients and their usage. For example, client B is connected to node 1 and uses service 1 for 6.62 usage and service 2 for 3.79 usage. Table 2 shows detailed information for each service in the network. In the sample network, three network services provide services through edge computing, and each service has its respective computing resource factors and traffic factors for each usage. For each usage 1, service 1 consumes 1.33 workloads of CPUs, 7.85 workloads of memory, 15.45 workloads of storage, and 2.49 Mbps of traffic per link. In addition, as factors that are not dependent on usage, base resources are consumed by 1.28 usage per node, and synchronization traffic is consumed by 2.49 Mbps per link.

We use the proposed algorithm to deploy the edge servers of these three services on the network. First, candidates for each service are generated and registered using Algorithm 1. In the case of service 0, the candidate generation process is relatively simple because it has only one client. According to Table 3, a total of five candidates for service 0 are generated, which means each case in which the server is deployed on one node among the five and is mapped to client C. When the server is deployed on node 2, the total traffic is the lowest, and when the server is deployed on other nodes, the total traffic is all the same at the high level. Obviously, all candidates have the same computing resource requirements, but the total expected traffic depends on the location of the server.

Table 4 shows the top 10 candidates for service 1. Because four clients use service 1, there are more considerations than for service 0. Candidate 0 is the best case and has the smallest total traffic of 4.99. Candidate 0 is a case in which three servers located in the nodes to which each client is connected are mapped to the corresponding clients, and there is no backhaul network traffic generated in the communication between the server and client; there is only synchronization traffic on the link between nodes 1 and 3 and the link between nodes 3 and 4. Therefore, the total expected traffic is 2.49×2=4.98. Candidate 1 represents a client mapping that can minimize traffic when there are two servers. Note that the role of the servers located in nodes 3 and 4 of candidate 0 is played by one server located at node 3 in candidate 1. The total usage of both servers of candidate 0 is the same as that of the single server of candidate 1, but the total computing resource requirements is larger in the case of candidate 0. This is due to the existence of the base resource, and it disproves the idea that a shared resource is efficient.

Table 5 shows the top 12 candidates for service 2. For service 2, the traffic factor per usage and synchronization traffic factor are low, so the difference in expected traffic between candidates is not large. To look at the total traffic calculation, we take candidate 8 as an example. The total traffic of candidate 8 is 1.78. Here, client B is mapped to the server at node 0 and clients C and E are mapped to the server at node 2. To obtain the expected traffic of this mapping $\tau_{m}$, the usage of the client, the traffic factor of the service, and the distance between the server and the client are multiplied for each client. Client B generates 3.7917 × 0.2149 × 1 ≈ 0.8148, client C generates 7.6354 × 0.2149 × 0 = 0, and client E generates 2.2116 × 0.2149 × 1 ≈ 0.4753 traffic load. In addition, it creates a minimum spanning tree to compute the synchronization traffic load for the candidate. In the current example, the gateway is omitted, so the tree is created with only one edge between node 0 and 2. The synchronization traffic load can be obtained as 0.4878 by multiplying the synchronization traffic factor and the number of edges of the tree. Thus, the total expected traffic of candidate 8 is 0.8148 + 0.4753 + 0.4878 = 1.7779 ≈ 1.78.

In the case of candidates 5 and 6, the number of servers and their locations are the same. In other words, this is a case where two different mappings exist for the same server assignment, and this was created because clients B and E have the same distance from both servers in the assignment. Therefore, when client B is mapped to node 2 and client E is mapped to node 3 and vice versa, a total of two candidates are registered.

After the candidate registration process, the final plan is generated by selecting the candidates. Two cases, in which the traffic weight ω (an input parameter of Algorithm 2) is 0.5 or 0, are presented. Here, a value of 0.5 means the vector angle and the traffic difference are considered equally, and 0 means only the vector angle is used. Table 6 shows the process of selecting candidates when ω is 0.5. First, all services use their prime candidates, and servers belonging to each candidate are deployed to all nodes except node 0. According to the results of the first trial, the server of candidate 0 of service 1 and the server of candidate 0 of service 2 are deployed to node 1. If deployed in this way, all nodes except node 2 can accommodate the computing resource requirements of the servers to be operated on those nodes. However, node 2 will not be able to satisfy the storage requirements, so server replacement by PVBP is required.

Table 7 shows the reconfiguration process according to the process in Table 6. In the first trial, node 2 becomes the reconfiguration node, and the vector angle and traffic difference for each server allocated to that node are computed. The vector angle and traffic difference of the servers belonging to candidate 0 of service 0 are 31.51 and 19.09, respectively, and the standardized values are 1.29 and 3.71, respectively. The vector angle and traffic difference of the server of service 2 are 30.40 and 0.01, respectively, and the standardized values are 1.18 and 0.98, respectively. The vector angle is slightly higher for the service 0 server, whereas the traffic difference is significantly lower for the service 2 server. Therefore, the server of service 2, which has a larger metric score, is replaced, and accordingly, candidate 0 of service 2 is removed, and candidate 1 of service 2 is newly added to the plan.

The second trial in Table 6 shows the resource status of each node after candidate 1 of service 2 is applied. Unfortunately, it is again confirmed that node 2 cannot meet the storage resource requirements. The second attempt in Table 7 shows the reconfiguration operation for the situation, and as before, the server of service 2 with a high metric score is selected as a replacement target. However, this time, it is confirmed that candidate 2, which is the immediately next candidate, is not selected, and candidate 12 is selected instead. This is the result of selecting a candidate that uses fewer resources of node 2 than candidate 1 from among the following candidates. For candidates 2 through 11, the requirements for node 2 are higher than for those of candidate 1. That is, it is obvious that when candidates 2 to 11 are selected, there is no difference in the resource requirements of node 2, so the same operation will be repeatedly performed. Returning to Table 6, according to the third trial, the candidate selection process ends because all nodes have satisfied the resource requirements. The total traffic load of the final plan is 7.58 Mbps, and the remaining resource ratio is 74.31%.

Table 8 shows the candidate selection process when ω is 0, that is, when traffic is not considered. In Table 9, as in the previous process, node 2 (which cannot meet the resource requirements) selects a server to be replaced, and the server of service 0 with the highest vector angle is selected. As a result, service 0 applies the next candidate, candidate 1, and it can be seen that all nodes have satisfied the resource requirements in the second trial. The total expected traffic is 25.04 Mbps, and the remaining resource ratio is 73.01%. Compared to the final plan that considers traffic, the remaining resources are reduced, but at the cost of an increase in traffic of about 330%.

In this example, the processes of creating and registering candidates for each service were presented, and the process of establishing the server deployment plan based on these candidate group was also introduced step by step. By adjusting the traffic weight value, the effect of changing the importance of traffic when selecting a server to be replaced was confirmed. It was indirectly confirmed that considering traffic can be of considerable help in resolving network congestion by sharply reducing the final total traffic volume without affecting the residual resource ratio.

## 4. Simulation Results

The proposed algorithm was evaluated by a simulation. In an actual environment, various network services each having a different requirement pattern exist, and various clients and network devices also exist. A simulation was performed to confirm that the proposed algorithm functions universally across these various environments.

### 4.1. Simulation Environment

Several aspects of the proposed scheme were evaluated through the simulation. The simulation results are analyzed by a comparison with random deployment, the first fit decreasing (FFD) scheme, and the previously proposed method [25].

**Figure 4:**
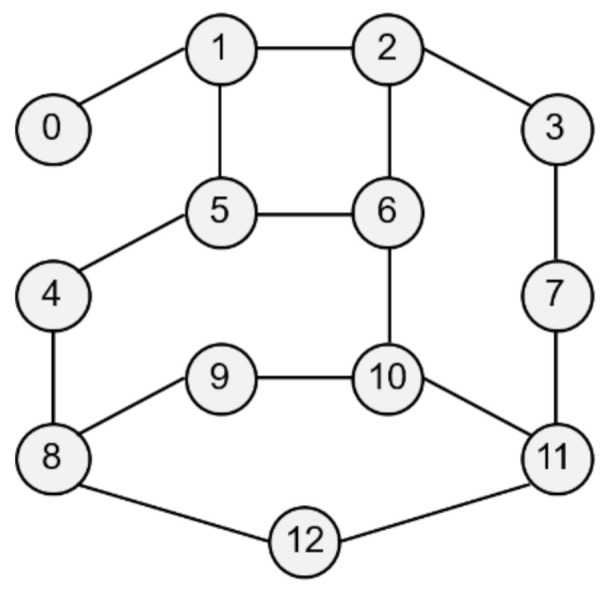
Sample network topology for the simulation.

The network topology shown in Figure 4 is used. The service characteristics and the usage per client are changed according to the purpose of simulation. Each network node, which is an edge node, has its own computing resource. It is assumed that there are several types of services in the network that respond to edge computing-based requests, and each service operates at least one edge server. A client, as a service user, is connected to the network through a single node and uses one or more services. The resource and traffic factors per usage are determined within a specific range, which can be treated as information that can be empirically obtained by system monitoring.

The default simulation parameters are presented below. In these simulations, it is assumed that the usage is much higher than in an actual environment. The resource capacities of an edge node are assumed to be $r^{cpu}\in \left[100,500\right],\;r^{mem}\in \left[100,1000\right],\;r^{sto}\in \left[1000,2000\right]$ (workload). We further assume that there are 10 types of services, and the computing resource factors per client usage are $f_{cpu}\in \left[0,\;10\right],\;f_{mem}\in \left[0,\;10\right],\;f_{sto}\in \left[0,\;100\right]$ and traffic factor is set to $f_{t}\in \left[0,\;3\right]$ (Mbps). The base resource of each service ranges between 0 to 3, and the synchronization traffic ranges between 0 to 5. It is assumed that there are a total of 10 clients, and usage of each client is a value between 0 to 10. In addition, the client uses the two services with a 20% probability. All random variables follow a uniform distribution.

The proposed algorithm was compared with the single-server VBP (SVBP) scheme, which was proposed in the previous study [25], FFD scheme, and a random (RND) scheme. SVBP is a method that generates a candidate with only a single server and then performs VBP considering only computing resources without traffic for the reconfiguration nodes. The FFD scheme is employed in the candidate selection process after candidate registration. FFD in a traditional bin packing problem is a greedy heuristic algorithm that puts specific items into bins in order. In this simulation, FFD was implemented so that the candidates are placed one by one from the first element in the list, in order from the first to the last service, and this is repeated until candidates for all services are normally deployed in the network. This approach is taken because the existing bin packing problem cannot support a diversity of servers within the candidate, and it is difficult to support various resource types [35,36]. Finally, RND is a method that selects and arranges a candidate randomly selected from the generated candidates. The proposed scheme was simulated while changing the traffic weight to 0, 0.25, 0.5, 0.75, and 1, and the results are referred to as PVBP-Q0, Q1, Q2, Q3, and Q4, respectively.

The reasons why FFD, which is a rather traditional scheme, was chosen as the algorithm to be compared is as follows. The proposed algorithm first configures a candidate list for each service, and establishes a final plan using the candidates. As such, the proposed algorithm essentially requires a candidate configuration step, so it is unreasonable to directly compare it with the approaches of other studies. Therefore, from the next step in which an ordered list of candidates for placement was configured, it was compared with the classic algorithm in the process of establishing the final plan. A comparison with this solution, which is somewhat traditional, but effectively improved for simulation, will be able to sufficiently verify the performance of the proposed preprocessing and deployment algorithms.

Many simulation parameters, such as the node resources, number of services, client location, and client usage, include randomness. Thus, to fully reflect this randomness, the simulations were performed at least 100,000 times for all conditions and the results are presented as arithmetic averages.

### 4.2. Simulation Results Analysis and Discussion

**Figure 5:**
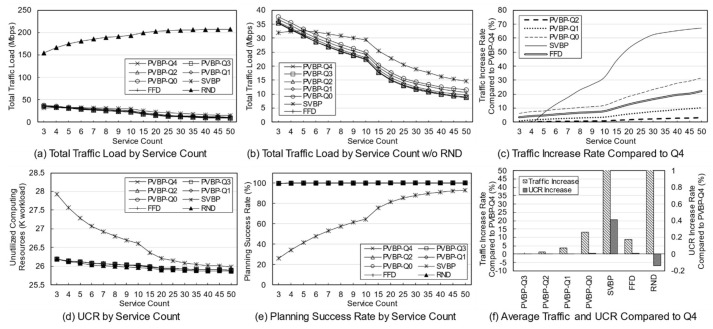
Key network metrics for the number of services according to the different management schemes and traffic weight.

First, a variation in the number of services was simulated to identify the effect of service diversity on the network. Figure 5 shows the simulation results. The number of clients is fixed at 10, and because each client uses two services with a 20% probability, a maximum of 20 services can be used. As the number of services increases, the probability that the used services overlap decreases. Because the simulation was performed assuming an overloaded situation, the methods may fail to establish the final deployment plan depending on the parameters or the algorithm. Failure means that edge servers cannot be deployed for any services when clients have a specific usage for a given service in a specific environment. In a general environment, the failure rate is not high, but in a situation where the usage is quite high, the failure rate varies greatly depending on the algorithm. An algorithm with a high success rate means that the reliability is high, and the edge network administrator can determine the use of the algorithm based on its reliability.

Figure 5a,b show the total traffic load according to the number of services. Here, the traffic load of the RND scheme remains at a very high level compared with those of other schemes. Obviously, the RND scheme is not designed to choose the best candidate. However, it is possible to indirectly grasp information such as the trend in the total traffic load of candidates occurring in a given situation. Figure 5b shows the total traffic load of the other schemes excluding RND. Overall, as the number of services increases, the total traffic load decreases. This means that in a situation where the number of clients and usage are fixed, an increase in the number of services means that there is a high possibility that a specific service will provide an edge service to only one specific client. For a service that is used only by one client, it is very effective to place the server on the node to which the client is connected, and the reason for creating candidates using more than one server does not apply. When the server of the service used by the client is deployed in the node to which the client is connected, the backhaul traffic load becomes zero. According to this result, increasing the diversity of services within the network means that the total traffic load on the network decreases.

Figure 5c shows the rate of total traffic load increase of other algorithms with respect to PVBP-Q4, which uses only the traffic difference in the main metric. As the number of services increases, the traffic control efficiency of other schemes decreases compared with that of PVBP-Q4. When there are 10 services, Q0 increases by 11.99%, FFD by 8.01%, and SVBP by 31.86% with respect to PVBP-Q4, and if there are 50 services, the increases are 31.47%, 22.35%, and 67.16%, respectively. This result means that the proposed candidate selection scheme reduces network congestion substantially better than the previous scheme, which considers only the number of computing resources and hops based on a single server. It also means that network congestion can be efficiently controlled through traffic weight adjustment. When the number of services is less than five, the result of SVBP is an abnormal number, which will be discussed later along with the success rate.

In the simulation, the efficiency of computing resource utilization is indicated using an index called the unutilized computing resources (UCR). UCR is the sum of unused computing resources, which includes resources that are wasted because of the exhaustion of other resources. For example, when the capacity of each of the CPU, memory, and storage of a specific node is 100, if the usage of the node becomes 100, 80, and 10, respectively, according to the deployment plan, the UCR of the corresponding node is 110. Relatively high UCR means that it provides edge service with fewer servers, which means that resources and operational efficiency are high. Because of its characteristics, UCR is more suitable for use as a relative index rather than a realistic value indicating the remaining amount of resources. Therefore, it is reasonable to use UCR for comparison with other schemes in terms of resource utilization, rather than using it for the direct analysis of a method’s performance.

Figure 5d shows the total UCR of all nodes according to the final plan of each algorithm. As the number of services increases, the change in UCR is only large for SVBP. This is because, in a situation in which the amount of remaining resources is relatively abundant (i.e., the density of the entities using the computing resource is low and there are few reconfiguration nodes), the resource usage amount of a specific service does not differ substantially in any node. Note that the computing resource utilization required for a particular service does not differ much among candidates. However, when two or more servers are operated, there is a difference that is equal to the additional base resource. Overall, for all schemes, as the number of services increases, the UCR decreases, which means that the number of services that provide edge service based on one server increases. The high UCR of SVBP is also addressed in the discussion on success rate.

Figure 5e shows the success rate of each algorithm. As mentioned earlier, the proposed algorithm determines the number of servers, deployment locations, and client mappings in a heuristic manner in a situation where service usage is relatively high, so there may be situations where it is impossible to obtain a final deployment plan. During the candidate selection process, failure occurs when it is determined that candidates for all services in a specific reconfiguration node have been exhausted. In other words, along with UCR, this index is an indicator of the comprehensive efficiency of computing resource utilization. Notable in this simulation is the success rate of SVBP. This scheme shows a success rate of about 26% when the number of services is three, and about 93% when the number of services is 50. For three services, SVBP failed 74,000 times in 100,000 simulations. When usage is concentrated on one service, it is often difficult to find a node that can provide it if only one edge server is configured. As the number of services increases, the average usage for one service naturally decreases, and it becomes easier to find a node where a server can be deployed. In contrast, because the other schemes, even RND, are based on candidates with multiple servers, the success rate is maintained at a 100% level.

The simulation was conducted 100,000 times for each algorithm for each number of services, and the failures were excluded when calculating the average values for traffic or computing resources. Suppose that scheme A selects candidates for three situations and the total traffic load for each one is 10, 20, and 30 Mbps. Moreover, assume scheme B obtains results of 15 Mbps for the first situation and fails for the subsequent two. The success rate of scheme A is 100% and the total traffic load is 20 Mbps on average, whereas the success rate of scheme B is 33% and the total traffic load is 15 Mbps on average. Such dramatic differences in success rates can lead to misleading simulation results. Therefore, in the simulation, the comparison is valid only when the success rate of each scheme is 90% or more, and the results with a rate of less than 90% are mentioned separately.

Figure 5f shows the average increase in traffic load and UCR with respect to PVBP-Q4 for each technique throughout the service diversity simulation. Q3, Q2, Q1, and Q0 mean that, respectively, 75%, 50%, 25%, and 0% of the traffic increase due to candidate replacement is considered when selecting candidates. In other words, compared with PVBP-Q4, the traffic loads in these methods are considered less and node computing resource utilizations are considered more. Compared to Q4, the traffic load of Q0 was 13.13% higher on average, whereas that of FFD was 8.80% higher and that of SVBP was 41.13% higher. The comparison data of SVBP was averaged only for values with a success rate of 90% or more to ensure a certain level of reliability in the discussion. In contrast, the increase in UCR was negligible: the increase in Q0 compared to Q4 was 0.01%, and that of SVBP was 0.41%. One reason may be that the total amount of usage generated by clients did not change even though the service diversity changed. Another reason may be that the number of servers for each service converges to one as the diversity of services increases, so that resource usage among candidates does not differ much.

**Figure 6:**
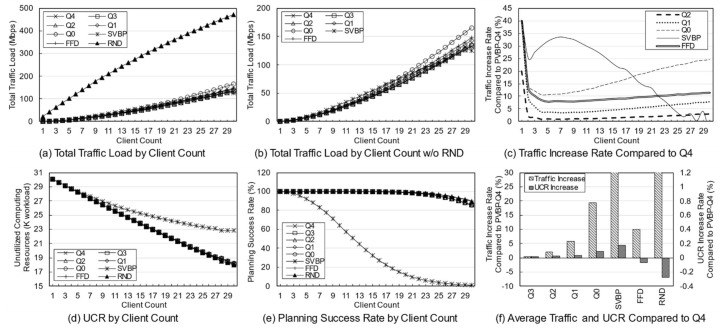
Key network metrics for the number of clients according to the different management schemes and traffic weight.

Figure 6 shows the simulation results according to the change in the number of clients. The number of services was fixed at 10 and the number of clients varied from 1 to 30. The client locations were randomly assigned in each simulation. Figure 6a,b show the amount of change in traffic load as the number of clients increases. In the simulation, the increase in the number of clients means an increase in the total usage in the network, so the total traffic load increases with the increase in usage. Figure 6c shows the increase in the traffic load of the techniques compared with that of PVBP-Q4. It can be seen that as the number of clients increases, the relative traffic loads of techniques such as Q1, Q0, and FFD increase. In the case of SVBP, the traffic load initially increases and then decreases. Eventually, the results oscillate. These are abnormal values caused by the low success rate, meaning that SVBP produces good results only under certain circumstances and fails otherwise.

Figure 6d shows the UCR according to the deployment plan for each algorithm, and Figure 6e shows the success rate of each algorithm. The UCRs decrease somewhat linearly as the number of clients increases. However, SVBP, as for its traffic load, yields a slope that differs from the results of other schemes, which is due to its low success rate. In the case of SVBP, when there are 12 clients, the deployment was successful only about 50% of the time, and when it exceeds 20, the success rate is less than 10%. As the usage increases, the probability of requests that cannot be serviced by the resources of single node will occur, leading to deployment failure. However, the success rate of the other schemes is about 90% even when computing resources in the whole network become insufficient due to an increase in the number of clients.

Figure 6f shows the average increase in traffic and unused resources with respect to PVBP-Q4 for each algorithm in the simulation with a variable number of clients. In the case of PVBP, as the traffic weight decreases, the traffic load of the plan increases by up to 20%. The traffic load of Q3 increased by 0.50%, Q0 by 20.21%, FFD by 10.16%, and SVBP by 31.09%. The amount of change in UCR is large compared to the results of the service diversity simulation, because the usage varies according to the number of clients. Q1 and Q0 had 0.04% and 0.1% resources left, respectively, but FFD and RND yielded results that used more resources than the results of Q4 by 0.1% and 0.3%, respectively. In other words, for FFD, which did not strictly consider traffic and resources, the traffic load increased, and the resource utilization efficiency decreased. If FFD is not based on a sorted candidate list, it will yield results close to the results of RND.

**Figure 7:**
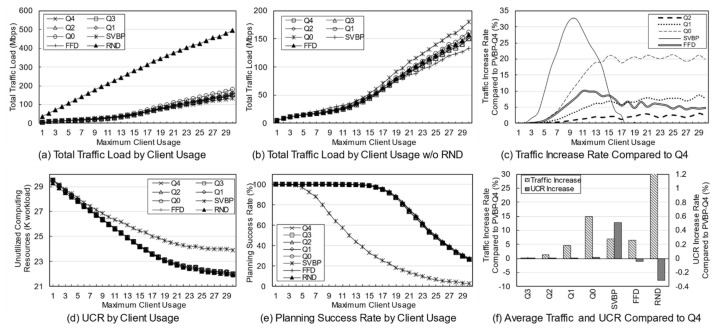
Key network metrics for the maximum client usage according to the different management schemes and traffic weight.

Figure 7 shows the simulation results according to the usage of each client. Unlike the previous simulation, the simulation was performed while fixing the number of clients to 10 and increasing the maximum usage per client from 1 to 30. To further clarify the difference, the situation when the simulation variable is at its maximum is given as an example. In the previous simulation, 30 clients each had a usage of 0 to 10, and in this simulation, 10 clients each had a usage of 0 to 30. Of course, as in the previous simulations, 20% of clients have 0 to 30 usage for each of the two services. In other words, the total usage of the same variable will be at the same level as the previous simulation, but the probability of intensive use increases from a service perspective, and the locality of clients is also slightly higher than the locality of the previous simulation.

Figure 7a,b show the total traffic load according to the usage of each client. The total traffic load is slightly higher than in the simulation related to the number of clients. Figure 7c shows the amount of traffic increase compared to PVBP-Q4 for each technique. Compared to the previous simulation, the increase is not high, and it can be seen that unpredictable results are obtained after the maximum usage of 15. This can be seen as a reliability problem stemming from the success rate of Q4.

Figure 7d shows the UCR result according to the maximum client usage. A comparison with the previous simulation confirms that the amount of remaining resources increases slightly. This is because the number of required servers decreases as the client locality slightly increases. Figure 7e shows the deployment success rate, which decreases more steeply than in the previous simulation. This is because, if a specific client uses a service with a high resource factor at a high level, it is difficult to find the node where the server mapped to that client will be placed. Figure 7f shows the average increase in traffic and unused resources compared to PVBP-Q4 for each scheme over the entire simulation. As for other simulations, the average value was calculated only for cases where the success rate was 90% or more. Compared with the previous simulation, the increase was reduced overall, but similar results were obtained.

The simulation results to analyze the impact of client locality when the maximum client usage is 2, 5, and 10 are discussed next. Client locality is quantified by the percentage of clients which are connected to one of the nodes in the network, and in the following simulation, the number of clients is set to 20. For example, if the locality is 30%, this means that six clients are connected to a specific node, and the remaining 14 clients are uniformly distributed across all nodes.

**Figure 8:**
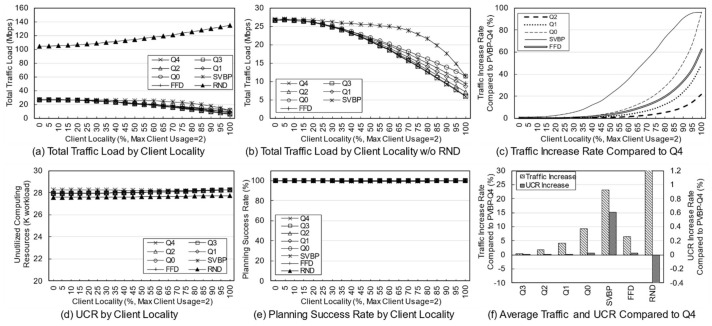
Key network metrics for the client locality when max client usage is two according to the different management schemes and traffic weight.

Figure 8 indicates the overall effect of locality on the network when the maximum client usage is 2. Figure 8a,b show the traffic load according to locality for each scheme. For all schemes except the RND scheme, the traffic load decreases as the locality increases. This is because, as more clients are connected to one node, the probability that each service will be based on a single server increases.

In addition, because the usage is low, there is a high possibility that the resource requirements of the server will not exceed the capacity of the node. In contrast, the traffic load of RND scheme increases, which means that the total number of candidates increases. This is because, as the number of clients connected to one node increases, the number of servers separated by the same distance within the same assignment increases, and thus a large number of mappings are generated.

Figure 8c shows the traffic load increase of other algorithms compared to PVBP-Q4 according to client locality when usage is low. As the degree of locality increases, the efficiency of traffic control of other algorithms decreases. Note that when the locality is 100%, the efficiency of SVBP and Q0 become almost equal. This is because both SVBP and Q0 select the server to be replaced based on the vector angle only. Moreover, because all the clients are connected to one node, high priority candidates will be composed of one server. Figure 8d–f also evaluate the performance of the proposed algorithm from the same perspective as the previous simulation. UCR does not change much depending on locality, and the success rate is nearly 100%.

**Figure 9:**
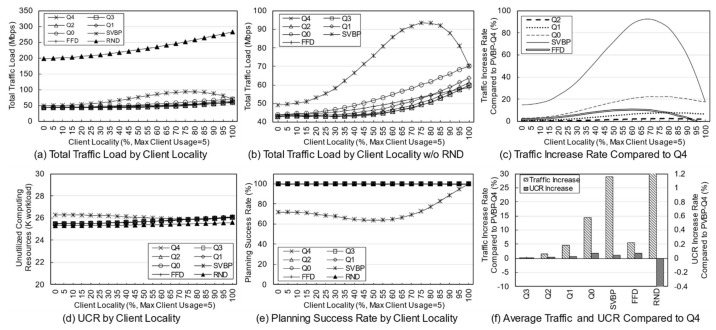
Key network metrics for the client locality when max client usage is 5 according to the different management schemes and traffic weight.

Figure 9 presents the effect of locality when the maximum client usage is 5, which is a moderate level. As shown in Figure 9a,b, the behavior is different from that of the previous simulation. That is, as locality increases, the traffic loads of all schemes except for SVBP increase. Because too much usage is concentrated at one point in the network, the server is deployed not only on the corresponding node, but also on nodes farther from it, which causes traffic in the backhaul network. In the SVBP scheme, the amount of traffic increases rapidly up to 75% locality, then decreases thereafter, and the traffic load is almost equal to Q0 at 100% locality. In Figure 9c, unlike in the previous simulation, the amount of traffic increase compared to Q4 is not very high. The peculiar thing is that the traffic control efficiency of FFD increases when the locality is high. The reason is that PVBP frequently performs candidate replacement to determine the final plan that has optimal traffic, and in this process, candidates for a specific service may be unnecessarily replaced. In a relatively simple situation with high locality, the approach of FFD, which is to search for candidates in one direction using only the candidate order, is slightly advantageous.

**Figure 10:**
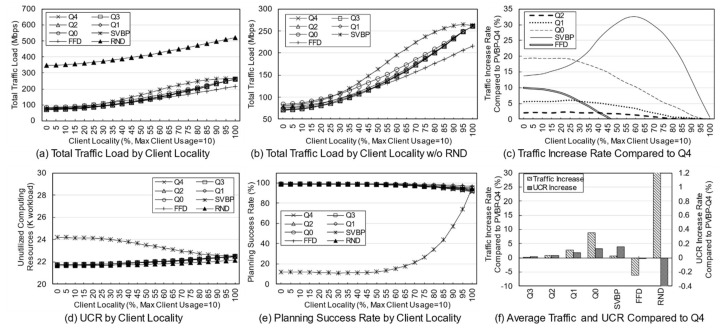
Key network metrics for the client locality when max client usage is 10 according to the different management schemes and traffic weight.

Figure 10 reveals the impact of locality when the client maximum usage is 10, which is a very high level. As the locality increases, the traffic load increases significantly, and when the locality is very high, the traffic control efficiency of other techniques instead increases compared to Q4. If the locality is very high, traffic is concentrated at one point in the network as in the previous simulation, so in this extreme case, the scheme considering the VBP-based resource ratio can be seen more as a way to select candidates with higher priority.

**Figure 11:**
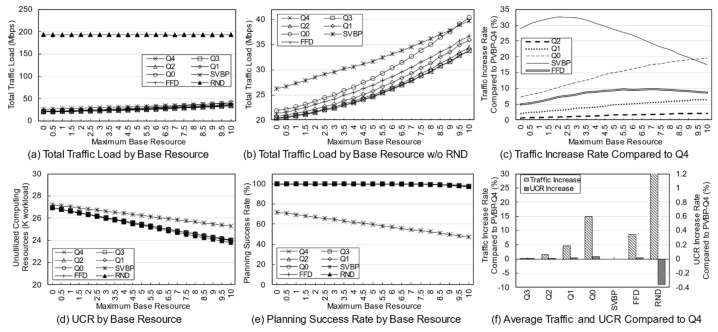
Key network metrics for the maximum base resource according to the different management schemes and traffic weight.

Figure 11 shows the simulation results that examine the impact of the base resources. The base resource is not dependent on usage, and the specified requirements according to the service characteristics exist on each node where the server of the service is placed. If the base resource is zero, the service can create multiple servers without adding any burden on computing resources.

In contrast, if the base resource is very high, such as 10, the benefits of operating two or more servers will be substantially offset. UCR also increases for all algorithms as the base resource increases. The success rate of SVBP gradually decreases as the resource requirements occupied by one server increases. Note that SVBP is not compared in Figure 11f because there is no result with a success rate of 90% or more in that simulation.

**Figure 12:**
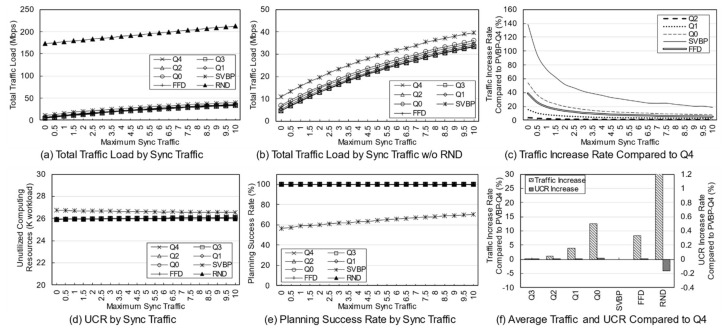
Key network metrics for the synchronization traffic according to the different management schemes and traffic weight.

Figure 12 shows the simulation results that reveal the effect of synchronization traffic. Synchronization traffic is traffic generated for information exchange between the servers and gateway nodes when the edge server is operated. If the synchronization traffic is 0, there is no need to consider distance when operating two or more servers. In contrast, as the value increases, the server location should be more carefully considered when generating candidates based on multiple servers. In Figure 12c, as the synchronization traffic increases, the traffic control efficiency of all other algorithms, including that of SVBP, approaches that of Q4. The reason for this is that as the synchronization traffic increases, the distance between the servers is inevitably smaller, so that the total traffic load of candidates with two or more servers increases. However, this only affects the candidate creation; it does not substantially affect the candidate selection of each scheme. Only the selected candidate increases the traffic load as much as the synchronization traffic. This can be confirmed by the constant difference between each scheme in Figure 12b.

## 5. Conclusions and Future Work

Edge computing can provide network services with low latency and real-time processing by operating cloud services at the network edge. Edge computing has numerous advantages such as low latency, localization, and network traffic distribution, but the resource management associated with its inherent hierarchical, distributed, and heterogeneous nature has become a significant challenge. Therefore, in this study, an algorithm that considers both the computing resources and network traffic load in the deployment of servers that provide edge services is proposed. Depending on the service characteristics and usage, the candidate deployments are generated based on factors that affect traffic load such as server location, number of servers, and server-client mapping. The final deployment plan is then established using a heuristic algorithm that selects the best candidates from among the candidates for various services. This selection uses a vector angle indicating the difference in resource ratios between the server and the node as well as the increase in traffic load when the next candidate is selected.

Various aspects of the proposed algorithm were compared with (i) the algorithm proposed in a previous study [25], which selected candidates that had a single server and considered only the vector angle, (ii) the FFD method, which selects candidates for each service in order, and (iii) a method using random candidate selection, through simulation. The effects of algorithm parameters (number of services, number of clients, usage per client, client locality, base resource, and synchronization traffic) on network performance indicators (total traffic load, total UCR, and deployment success rate) were analyzed. In addition, the effect of the weight accorded to traffic load on the placement result was analyzed from various perspectives.

In future, we plan to further refine the system model. Based on the findings in this study, we will apply a linear optimization method by establishing an objective function through a problem statement for generating and selecting candidates. In addition, we plan to diversify the form of collaboration between edge servers. In addition to cloning, this may include hierarchical server configurations, pipelining configurations between servers performing different operations, and hybrid servers. Through this, we will continue to contribute to the main topic of edge computing: resource management.

## Figures and Tables

**Table 1:** Symbols used to describe the algorithms.

Symbol	Meaning
V	The set of network services, v∈V
N	The set of edge nodes, n∈N
C	The set of network clients, c∈C
C	The list for the edge server placement candidates, 𝕔∈C
S	The set of edge servers, s∈S
A	The set of assignments that consists of the sets of key-value pairs, a∈A
M	The set of mapping information that consists of the sets of key-value pairs, m∈M
λ	The number of servers considered when configuring candidates
τ	The expected traffic when the candidate is adopted
R	The set of resources used by the candidate, r∈R
u	The client usage
f	The factor of specific resource per client usage of the service
ω	The weighted value for traffic as a metric
P	The final deployment plan, set of selected candidates by service
α	The vector angle between the edge server and the edge node

**Table 2:** Details of the example network services.

id	CPU Factor	Memory Factor	Storage Factor	Traffic Factor	Base Resource	Sync Traffic
0	1.30	8.28	92.11	2.72	2.83	3.36
1	1.33	7.85	15.45	2.49	1.28	2.49
2	3.08	9.67	94.05	0.21	1.48	0.49

**Table 3:** Service 0 candidate list.

No.	Total Traffic	Planned Servers					
		Node	Clients	Usage	CPU	Memory	Storage
0	0.00	2	*C*	7.01	12.80	81.40	905.94
1	19.09	0	*C*	7.01	12.80	81.40	905.94
2	19.09	1	*C*	7.01	12.80	81.40	905.94
3	19.09	3	*C*	7.01	12.80	81.40	905.94
4	19.09	4	*C*	7.01	12.80	81.40	905.94

**Table 4:** Service 1 candidate list.

No.	Total Traffic	Planned Servers					
		Node	Clients	Usage	CPU	Memory	Storage
0	4.98	1	A, B	8.71	13.28	78.43	154.39
		3	D	4.67	7.91	46.73	91.98
		4	F	3.90	6.88	40.63	79.98
1	12.21	1	A, B	8.71	13.28	78.43	154.39
		3	D, F	8.57	13.10	77.33	152.22
2	14.70	1	A, B	8.71	13.28	78.43	154.39
		2	F	3.90	6.88	40.63	79.98
		3	D	4.67	7.91	46.73	91.98
3	16.64	1	A, B	8.71	13.28	78.43	154.39
		4	D, F	8.57	13.10	77.33	152.22
4	16.64	1	A, B	8.71	13.28	78.43	154.39
		2	D	4.67	7.91	46.73	91.98
		4	F	3.90	6.88	40.63	79.98
5	16.64	1	A, B, D	13.39	19.50	115.13	226.63
		4	F	3.90	6.88	40.63	79.98
6	23.86	1	A, B	8.71	13.28	78.43	154.39
		2	D, F	8.57	13.10	77.33	152.22
7	23.86	1	A, B, D	13.39	19.50	115.13	226.63
		2	F	3.90	6.88	40.63	79.98
8	24.21	3	A, B, D	13.39	19.50	115.13	226.63
		4	F	3.90	6.88	40.63	79.98
9	26.70	2	A	2.10	4.49	26.49	52.14
		3	B, D	11.29	16.71	98.67	194.23
		4	F	3.90	6.88	40.63	79.98
10	26.70	2	A, B	8.71	13.29	78.43	154.39
		3	D	4.67	7.91	46.73	91.98
		4	F	3.90	6.88	40.63	79.98

**Table 5:** Service 2 candidate list.

No.	Total Traffic	Planned Servers					
		Node	Clients	Usage	CPU	Memory	Storage
0	0.96	1	B	3.79	16.24	50.97	495.71
		2	C, E	9.85	34.89	109.53	1065.21
1	0.98	1	B	3.79	16.24	50.97	495.71
		2	C	7.64	28.08	88.14	857.21
		4	E	2.21	11.37	35.69	347.10
2	1.29	2	B, C, E	13.64	46.57	146.19	1421.83
3	1.30	2	B, C	11.43	39.76	124.81	1213.83
		4	E	2.21	11.37	35.69	347.10
4	1.45	1	B	3.79	16.24	50.97	495.71
		2	C	7.64	28.08	88.14	857.21
		3	E	2.21	11.37	35.69	347.10
5	1.78	2	C, E	9.85	34.89	109.53	1065.21
		3	B	3.79	16.24	50.97	495.71
6	1.78	2	B, C	11.43	39.76	124.81	1213.83
		3	E	2.21	11.37	35.69	347.10
7	1.78	2	C	7.64	28.08	88.14	857.21
		3	B, E	6.00	23.05	72.36	703.71
8	1.78	0	B	3.79	16.24	50.97	495.71
		2	C, E	9.85	34.89	109.53	1065.21
9	1.79	0	B	3.79	16.24	50.97	495.71
		2	C	7.64	28.08	88.14	857.21
		4	E	2.21	11.37	35.69	347.10
10	1.79	2	C	7.64	28.08	88.14	857.21
		3	B	3.79	16.24	50.97	495.71
		4	E	2.21	11.37	35.69	347.10
11	2.27	0	B	3.79	16.24	50.97	495.71
		2	C	7.64	28.08	88.14	857.21
		3	E	2.21	11.37	35.69	347.10
12	2.59	1	B, C, E	13.64	46.57	146.19	1421.83

**Table 6:** Weight 0.5 PVBP.

Trial	Node	Services (Cand. Idx)	CPU (Usage/Cap.)	Memory (Usage/Cap.)	Storage (Usage/Cap.)
1	0	-	0.00/458	0.00/276	0.00/1664
	1	1 (0), 2 (0)	29.52/287	129.40/503	650.10/1699
	2	0 (0), 2 (0)	47.69/423	190.93/997	1971.15/1462*
	3	1 (0)	7.91/167	46.73/497	91.98/1414
	4	1 (0)	6.88/394	40.63/352	79.98/1609
2	0	-	0.00/458	0.00/276	0.00/1664
	1	1 (0), 2 (1)	29.52/287	129.40/503	650.10/1699
	2	0 (0), 2 (1)	40.88/423	169.54/997	1763.15/1462 *
	3	1 (0)	7.91/167	46.73/497	91.98/1414
	4	1 (0), 2 (1)	18.25/394	76.32/352	427.07/1609
3	0	-	0.00/458	0.00/276	0.00/1664
	1	1 (0), 2 (12)	59.86/287	224.63/503	1576.22/1699
	2	0 (0)	12.80/423	81.40/997	905.94/1462
	3	1 (0)	7.91/167	46.73/497	91.98/1414
	4	1 (0)	6.88/394	40.63/352	79.98/1609

* Resource request exceeding the node capacity.

**Table 7:** Reconfiguration processes with weight 0.5 PVBP.

Trial	Node	Services	Vector Angle	Standard V.A.	Traffic Diff	Standard T.D.	Metric	Result
1	2	0(0)	31.51	1.29	19.09	3.71	−1.21	Hold
		2(0)	30.40	1.18	0.01	0.98	0.10	To 1
2	2	0(0)	31.51	1.46	19.09	3.71	−1.13	Hold
		2(1)	30.40	1.34	0.31	1.12	0.11	To 12

**Table 8:** Weight 0 PVBP.

Trial	Node	Services	CPU (Usage/Cap.)	Memory (Usage/Cap.)	Storage (Usage/Cap.)
1	0	-	0.00/458	0.00/276	0.00/1664
	1	1 (0), 2 (0)	29.52/287	129.40/503	650.10/1699
	2	0 (0), 2 (0)	47.69/423	190.93/997	1971.15/1462 *
	3	1 (0)	7.91/167	46.73/497	91.98/1414
	4	1 (0)	6.88/394	40.63/352	79.98/1609
2	0	0 (1)	12.80/458	81.40/276	905.94/1664
	1	1 (0), 2 (0)	59.86/287	224.63/503	1576.22/1699
	2	2 (0)	34.89/423	109.52/997	1065.21/1462
	3	1 (0)	7.91/167	46.73/497	91.98/1414
	4	1 (0)	6.88/394	40.63/352	79.98/1609

* Resource request exceeding the node capacity.

**Table 9:** Reconfiguration processes with weight 0 PVBP.

Trial	Node	Services	Vector Angle	Standard V.A.	Traffic Diff	Standard T.D.	Metric	Result
1	2	0(0)	31.51	1.29	19.09	3.71	1.29	To 1
		2(0)	30.40	1.18	0.01	0.98	1.18	Hold

## Data Availability

Not applicable.
